# The Role of microRNAs in the Pathogenesis of Herpesvirus Infection

**DOI:** 10.3390/v8060156

**Published:** 2016-06-02

**Authors:** Diogo Piedade, José Miguel Azevedo-Pereira

**Affiliations:** Host-Pathogen Interaction Unit, iMed.ULisboa, Faculdade de Farmácia, Universidade de Lisboa, 1649-003 Lisboa, Portugal; diogo.piedade.92@gmail.com

**Keywords:** microRNAs, herpesvirus, pathogenesis, latency, oncogenesis, immune evasion

## Abstract

MicroRNAs (miRNAs) are small non-coding RNAs important in gene regulation. They are able to regulate mRNA translation through base-pair complementarity. Cellular miRNAs have been involved in the regulation of nearly all cellular pathways, and their deregulation has been associated with several diseases such as cancer. Given the importance of microRNAs to cell homeostasis, it is no surprise that viruses have evolved to take advantage of this cellular pathway. Viruses have been reported to be able to encode and express functional viral microRNAs that target both viral and cellular transcripts. Moreover, viral inhibition of key proteins from the microRNA pathway and important changes in cellular microRNA pool have been reported upon viral infection. In addition, viruses have developed multiple mechanisms to avoid being targeted by cellular microRNAs. This complex interaction between host and viruses to control the microRNA pathway usually favors viral infection and persistence by either reducing immune detection, avoiding apoptosis, promoting cell growth, or promoting lytic or latent infection. One of the best examples of this virus-host-microRNA interplay emanates from members of the *Herperviridae* family, namely the herpes simplex virus type 1 and type 2 (HSV-1 and HSV-2), human cytomegalovirus (HCMV), human herpesvirus 8 (HHV-8), and the Epstein–Barr virus (EBV). In this review, we will focus on the general functions of microRNAs and the interactions between herpesviruses, human hosts, and microRNAs and will delve into the related mechanisms that contribute to infection and pathogenesis.

## 1. Introduction

MicroRNAs (miRNAs) are small, approximately 22-nucleoide-long, non-coding RNAs that regulate gene expression [1,2,3,4]. They were discovered in 1993 in studies into the development of *Caenorhabditis* *elegans* [5,6]. Gene expression control exerted by miRNAs is post-transcriptional as miRNAs regulate mRNA translation and stability in the cytoplasm [7,8,9]. There are over 1000 miRNAs encoded in the human genome, and they are predicted to regulate over 60% of our genes [10,11]. Hence, miRNAs seem to participate in virtually every cellular process, and changes in their expression are present in human pathologies [12,13,14,15].

Interactions between viruses and the RNA interference (RNAi) pathway, which includes miRNA and small interfering RNA (siRNA) pathways, were first described during viral infection of plants and afterwards tested in *Drosophila* systems [16,17]. Despite evidence in plants and insects, it was thought that this pathway was not antiviral in mammalian cells due to interferon system activation by viral dsRNA [18]. It was only with the discovery that siRNAs did not induce interferon machinery [19] and that the interplay between viruses and RNAi machinery was considered.

To date, several types of mutual interference between a virus and the host cell’s miRNA machinery have been described. Viruses can (i) avoid cellular miRNAs targeting viral mRNAs [20]; (ii) block or impair the miRNA pathway by interacting with some key proteins [21,22]; (iii) synthesize their own miRNA to generate a more favorable cellular environment or to regulate their own mRNAs [23,24]; or (iv) make use of cellular miRNAs to their favor [25]. On the other hand, cells are also able to target viral mRNAs with endogenous miRNAs [26,27,28]. This interplay between the host-cell’s miRNAs and viruses raises a complex equilibrium where, most of the time, viruses have the advantage and are able to escape the immune response and make use of cellular miRNAs in order to complete their replication cycle. However, several degrees of uncertainty are present; the experimental challenges raised by miRNA target prediction and functional validation is one of the most important issues that should be highlighted, motivating the development of methods to effectively assess miRNA functions.

*Herpesviridae* is a family of large nuclear DNA viruses (herpesviruses). These viruses are able to establish lifelong infection in their host due to interchange between lytic (productive) and latent (non-productive) infection [29,30]. There are eight human herpesvirus that can be subdivided in three subfamilies: (i) *Alphaherpesvirinae*; (ii) *Betaherpesvirinae*; and (iii) *Gammaherpesvirinae* [30]. Herpesvirus infection is associated with a large array of clinical manifestations spanning from skin/mucosal lesions to several malignancies. The focus of this review will cover all three subfamilies but will mainly discuss herpesviruses that are known to encode miRNAs.

Among all viral miRNAs already identified, the majority is encoded and expressed by herpesviruses [24,31]. Human herpesvirus confirmed to encode miRNAs are: the herpes simplex virus type 1 and type 2 (HSV-1 and HSV-2) both belonging to the *Alphaherpesvirinae* subfamily [32], human cytomegalovirus (HCMV) from the *Betaherpesvirinae* subfamily [33], and, finally, the Epstein–Barr virus (EBV) and human herpesvirus 8 (HHV-8), also known as Kaposi’s sarcoma-associated herpesvirus (KSHV), both from *Gammaherpesvirinae* subfamily [34,35].

Herpesviruses’ miRNAs are able to target both cellular and viral mRNAs. Host miRNA-targeted mRNAs are mostly related to cell proliferation regulation, apoptosis, and host immunity. Viral mRNA targets present various functions, and viral miRNAs seem to have a role in evading host immunity and regulating lytic and latent infection [30,36,37,38,39].

## 2. Overview of miRNA Biogenesis

miRNA biogenesis begins in the nucleus with the transcription of miRNA genes by RNA polymerase II [40]. miRNA genes are often found in clusters that are transcribed together in intergenic regions, and they have their own regulatory sequences in both sense and antisense orientations [41]. These transcripts, known as primary-miRNAs (pri-miRNAs), contain a 5′-end cap and a poly-A tail sequence.

Pri-miRNAs are processed to functional miRNAs in two steps catalysed by two enzymes of the RNase III family, Drosha and Dicer. The first step is mediated by the Drosha and DiGeorge syndrome critical region gene-8 (DGCR8) complex [42] and occurs in the nucleus. During this step, pri-miRNAs are processed to precursor miRNAs (pre-miRNAs) that are exported from the nucleus by Exportin-5 [43,44,45]. The second step takes place in the cytoplasm where pre-miRNAs are further processed by Dicer [46,47]. This final processing step produces a 21- to 25-nucleotide mature dsRNA that is ready to be loaded into a RNA-induced silencing complex (RISC) [48], forming the miRNA-induced RISC (miRISC) where miRNA–mRNA binding occurs (further reviewed in [49]).

miRNAs recognize and interact with the corresponding mRNA by base-pair complementarity. Generally, animal cells’ miRNAs pair imperfectly with target mRNAs in the 3′ untranslated region (3′UTR). In order to effectively suppress their target mRNAs, miRNAs must be perfect and contiguously complementary in their nucleotides 2–8 at the 5′end; this region is called the ‘seed’ region and it nucleates the interaction [50,51]. However, additional 3′ pairing increases the miRNA-induced repression [51], suggesting that multiple miRNAs are needed to regulate target expression if miRNA complementary is only based in the 5′ seed region. The need for a synergistic binding of distinct miRNAs may constitute an additional mechanism of miRNA-mediated inhibition of translation in animal cells [50]. miRNAs can repress mRNA in two distinct ways: they could inhibit translation, or they can destabilize mRNAs. The most recent studies suggest that these two mechanisms occur in sequence [52,53,54,55,56], indicating that destabilization of target mRNA is the last and predominant step in mRNA repression [52,57].

## 3. Alphaherpesvirus (HSV-1 and HSV-2) and microRNAs

### 3.1. Targeting Viral Transcripts to Maintain Latency

Alphaherpesviruses, especially both serotypes of human simplex virus, type 1 and type 2 (HSV-1 and HSV-2), infect humans by contact of oral or genital mucosa with viral particles. From the mucosa, the virus migrates and establishes a latent infection in sensory or autonomic neurons near the primary site of infection [32,58]. During latent infection by HSV, only a non-coding viral RNA known as latency-associated transcript (LAT) is expressed [59]. Although not essential for latency establishment, maintenance, or reactivation [60], LAT plays an important role in these processes probably by interfering with other viral transcripts due to LAT-encoded miRNAs [61,62,63,64,65].

**Figure 1:**
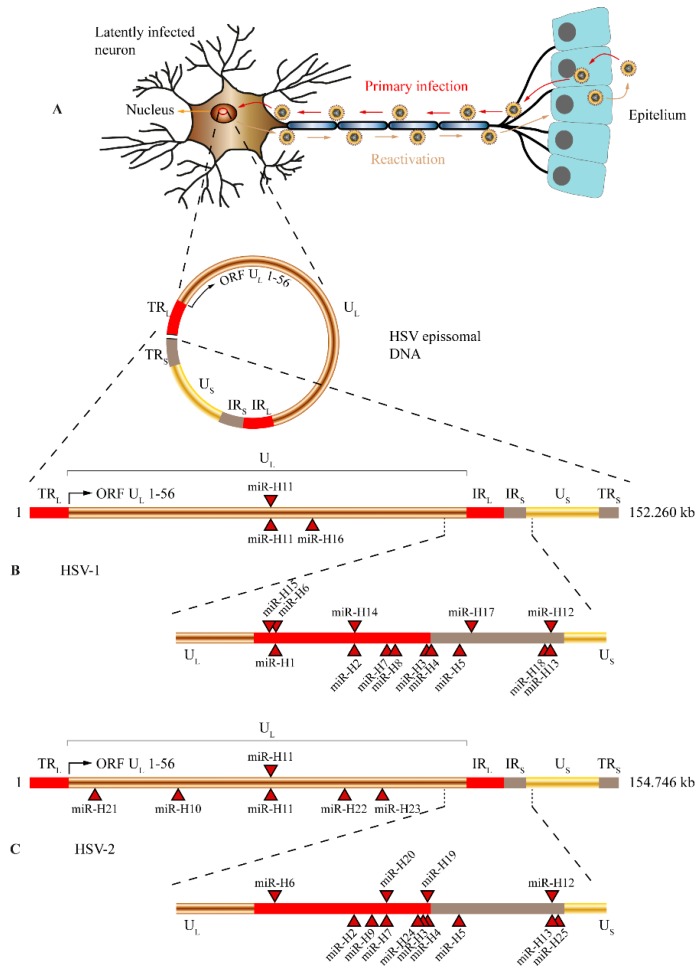
Viral genomic DNA structure and location of microRNAs (miRNAs) encoded by herpes simplex virus type 1 and type 2 (HSV-1 and HSV-2). (**A**) Episomal DNA in a latently infected neuron. After primary infection, HSV-1 or HSV-2 virions migrate from oral or genital mucosa to a sensitive neuron that enervates those tissues establishing a latent infection. The migration through neuronal dendrites occurs by a retrograde microtubule-associated transport, allowing HSV to reach nerve cell body. As a response to several exogenous or endogenous stimuli, the latent virus is reactivated and viral particles are anterograde transported to the mucosa where they infect epithelial cells allowing viral transmission. During latency, the HSV genome is maintained as a circular episome (represented as a red circle inside the nucleus). The structure and prototypic arrangement of the HSV episome is presented. U_L_ (copper) and U_S_ (gold) represent the long and short components of the viral genome. TR_L_ and IR_L_ (in red) represent the repeat sequences flanking U_L_ and TR_S_, and IR_S_ (in light brown) represent the repeat sequences flanking U_S_. Genomic location of miRNAs encoded by HSV-1 (**B**) and HSV-2 (**C**). A linear representation of both HSV-1 and HSV-2 genomes are shown, and the locations of miRNA precursors encoded by each virus are denoted by red triangles. The regions of the internal repeat sequences (IR_L_ and IR_S_) are expanded in the bottom of each genome. miRNAs shown below the line representing viral double-strand DNA are transcribed in an antisense direction (from left to right), while those shown above the line are transcribed in the opposite orientation.

HSV-1 encodes 18 stem-loops that result in 27 mature miRNA sequences [61,63,64,66,67,68,69], while HSV-2 also encodes for 18 stem-loops but only produce 24 mature miRNAs [62,65,66] (Figure 1). The first report of a HSV-1 miRNA was in 2006 [61]; since then, several studies have attempted to unravel the functions of HSV-1 miRNAs [62,63,67,70,71]. Despite these efforts, the functions of the majority of HSV-1 miRNA functions remain unknown. Even so, the functions that have already been revealed point to a relevant role of HSV-1 miRNAs in the regulation of latency. One good example is the targeting of the infected cell polypeptide 4 (ICP4) protein by hsv1-miR-H6 [70]. ICP4 protein is an immediate early gene of HSV-1 that upregulates early and late genes of HSV-1 and downregulates LAT, driving the virus towards lytic infection [62,67,70]. Therefore, hsv1-miR-H6 seems to play an important role in latency maintenance of HSV-1 [70]. Another HSV-1 miRNA that potentially regulates latency is hsv1-miR-H2. This viral miRNA targets ICP0 protein, other immediate early gene that acts as an activator of immediate early, early, and late genes of HSV-1 [62,67]. ICP0 has a major role in lytic infection and reactivation, and its expression promotes the entry of HSV-1 in replication cycle [67,72]. Another viral protein targeted by HSV-1 miRNAs is ICP34.5, an important lytic neurovirulence factor. ICP34.5 is targeted by two viral miRNAs, hsv1-miR-H3 and hsv1-miR-H4, encoded in an antisense orientation to the viral factor [62,63,67]. Altogether, these results indicate that hsv1-miR-H6, hsv1-miR-H2, hsv1-miR-H3, and hsv1-miR-H4 seem to play a role in the establishment and maintenance of latency while evading host immune surveillance.

Despite the fact that most identified targets of HSV-1 miRNAs are virus-encoded, hsv1-miR-H27 targets cellular transcriptional repressor Kelch-like 24 (KLHL24), which inhibits transcriptional efficiency of viral immediate early and early genes. Thus, opposite to other viral miRNAs, hsv1-miR-H27 seems to have an important role in immune evasion, viral replication, and proliferation [68]. Interestingly, HSV-2 and HSV-1 share a great homology between mRNA sequences [32,66]. Unsurprisingly, HSV-2 miRNAs sharing homology with HSV-1 miRNAs have similar functions, as it is the case of hsv2-miR-H2 and hsv2-miR-H3/4, which target ICP0 and ICP34.5, respectively. Therefore, HSV-2 miRNAs contribute to latency and immune evasion in an almost identical way to HSV-1 miRNAs [62,65,66,71].

### 3.2. miRNAs Patterns of Accumulation in Alphaherpesvirus Support Viral miRNAs Predicted Functions

Given the apparent role of HSV miRNAs in the establishment of latency, the pattern of accumulation of HSV-1 miRNAs in infected cells was evaluated in order to confirm differential expression of miRNAs in lytic and latent infection [58,67]. Not surprisingly, patterns of viral miRNA expression support the proposed functions for these viral transcripts. All latency-associated viral miRNAs, namely, hsv1-miR-H6, hsv1-miR-H2, hsv1-miR-H3, and hsv1-miR-H4, were upregulated in cells harvested from latently infected murine ganglia [58]. Interestingly, hsv1-miR-H5 and hsv1-miR-H7 also shown a similar pattern of expression, suggesting a potential role for these miRNAs in the latency of HSV-1 [58]. Moreover, several other viral miRNAs levels were significantly increased upon HSV-1 reactivation. Among these is hsv1-miR-H27, supporting its role in efficient replication and proliferation [58]. Besides hsv1-miR-H27, hsv1-miR-H15, hsv1-miR-H17, hsv1-miR-H18, and hsv1-miR-H26 also had a significant increase in their levels, suggesting a potential role in HSV-1 reactivation [58].

### 3.3. Cellular miRNAs Are Involved in Infection and Latency of Alphaherpesvirus

Another aspect worth mentioning regarding HSV-1 and miRNAs are the cellular miRNAs that interfere with viral replication. In fact, cellular miRNAs are known to be engaged in several antiviral functions [26], and HSV-1 infection is no exception. Cellular miRNA miR-101 targets the 3′ untranslated region of mitochondrial ATP synthase subunit beta (ATP5B), a cellular protein that plays a role in viral infection, reducing HSV-1 replication [73]. Therefore, this miR-101/ATP5B axis acts as a cellular defence that avoids lytic replication of HSV-1 and may contribute to latency [73]. Interestingly, miR-23a was found to facilitate HSV-1 replication by targeting interferon regulatory factor 1 (IRF1) and inhibiting the interferon pathway, an antiviral innate immune pathway [74]. This complex interaction is not yet fully understood, and the mechanisms by which HSV-1 induces miR-23a expression are not clear [74]. Another cellular miRNA altered by HSV-1 infection is miR-146a [75]. HSV-1 induces the pro-inflammatory miR-146a. This cellular miRNA is known to target complement factor H and to induce key elements of the arachidonic acid cascade. Thus, this mechanism provides an HSV-1 way to evade the complement while contributing to an Alzheimer-type neuropathological change [75].

In conclusion, alphaherpesviruses evolved in a way that allows them to establish lifelong latency in host neurons. Viral miRNAs and the interplay between cellular miRNAs and HSV-1 and HSV-2 seem to play an important role in the events related to latency, such as establishment, maintenance, and reactivation of the virus. In addition, miRNAs have shown to be linked with pathological events related to HSV infection. Therefore, HSV miRNAs may constitute a new and different approach to antiviral therapy.

## 4. Human Cytomegalovirus microRNAs

**Figure 2:**
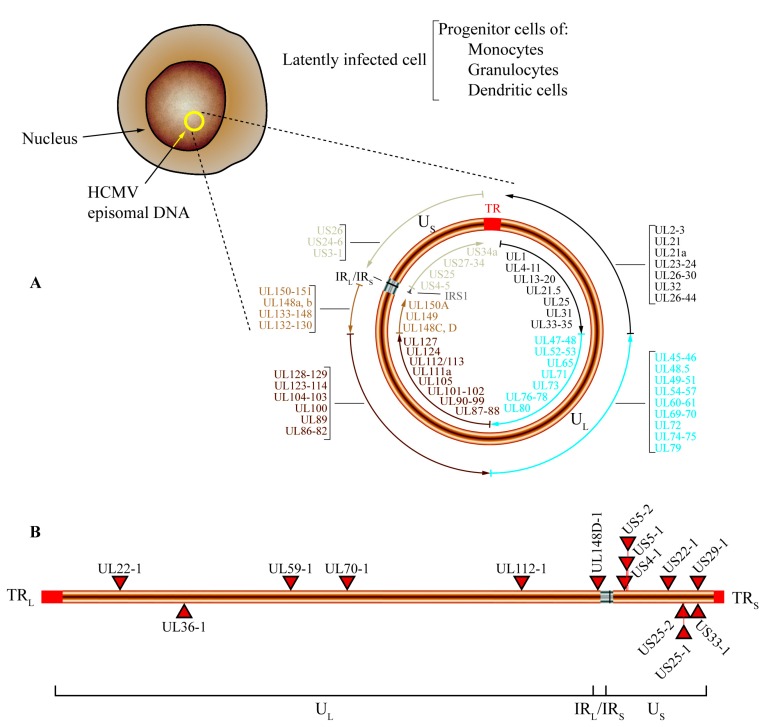
Genome organization and location of miRNA encoded by human cytomegalovirus (HCMV). (**A**) After primary infection HCMV establishes a latent infection in progenitor cells of monocytes, dendritic cells, and granulocytes. During latency, the HCMV genome is maintained as a circular episome (represented as a yellow circle inside the nucleus). The general structure of episomal DNA is presented, and the relative positions of the open reading frames (ORFs) are shown. U_L_ and U_S_ represent the long and short components of the viral genome, TR (red) denotes the terminal repeats, and IR_L_/IR_S_ (grey) represents the internal repeats of the long and short components of the HCMV genome. The outer circular arrows refer to the ORFs that are transcribed in antisense orientation, while the inner circular arrows refer to those that are transcribed in the opposite orientation. To facilitate the representation of the different ORFs and their relative location, the outer and inner circular arrows are presented in different colors; the black circular arrows represent those ORFs encoded between 12 and 58 kb of the HCMV genome, the blue circular arrows represent ORFs encoded between 59 and 118 kb, the dark brown circular arrows denote the ORFs encoded between 119 and 177 kb, the light brown circular arrows refer to the ORFs located between 178 kb and the IRL/IRS region, and finally the light grey circular arrows represent the ORFs that are encoded after the IRL/IRS region; (**B**) Genomic location of miRNAs encoded by HCMV. A linear representation of the HCMV genome is shown and the locations of miRNA precursors are denoted by red triangles. miRNAs shown below the line representing viral double-strand DNA are transcribed in antisense direction (from left to right), while those shown above are transcribed in the opposite orientation.

Human cytomegalovirus (HCMV) belongs to the *Betaherpesvirinae* subfamily and characteristically establishes latent infection upon resolution of acute infection [76]. HCMV infection can cause increased morbidity and mortality in immunosuppressed patients such as transplant recipients and human immunodeficiency virus (HIV)-infected patients [76]. Furthermore, HCMV primary infection during pregnancy is also associated with congenital infection, leading to severe birth defects and neonatal pathologies. The HCMV miRNAs can target both viral and host genes, playing a key role in latency and replication of HCMV [77]. There is contradictory evidence regarding the amount of miRNAs expressed by HCMV. While miRBase [11] data point to 15 stem-loop precursors and 26 mature miRNA sequences, the latest reports only account for 21 mature miRNA sequences originating from 14 precursors [77] (Figure 2). Contrary to other herpesvirus subfamilies, HCMV miRNAs are not clustered in regions associated with latent transcripts; instead, HCMV miRNAs are dispersed throughout the viral genome as single miRNAs or small clusters [78,79,80,81] (Figure 2).

### 4.1. HCMV miRNAs Target Cellular Genes to Evade Immune System and Control Cell Cycle as Well as Vesicle Trafficking

HCMV miRNAs were predicted to target several cellular pathways—in particular, those related with immune response, cell cycle control, and vesicle trafficking [33,77]. Regarding immune evasion, HCMV miRNAs were confirmed to target multiple pathways, ranging from cell recognition and proliferation to cytokine secretion. For example, the viral miRNA hcmv-miR-UL112 targets the major histocompatibility complex class-I related chain B (MICB) reducing natural-killer (NK) group 2, member D (NKG2D) recognition, helping the evasion of HCMV from NK cells [82]. Interestingly, the hcmv-miR-UL112 binding site has been shown to overlap the one from cellular miRNA hsa-miR-373, preventing site mutation by the host [83]. Additionally, this viral miRNA was found to act synergistically with another cellular miRNA, hsa-miR-376a. Both cellular and viral miRNA were able to decrease MICB levels and NK-mediated cell killing, suggesting that HCMV may have evolved in a way that allows cooperation with host miRNAs and the targeting of highly conserved sequences in 3′UTR of host mRNA [83]. Moreover, hcmv-miR-UL112-1 was reported to target interleukin-32 which is critical for innate and adaptive immune response [84]. Another viral miRNA that targets cellular mRNA related to immune evasion is hcmv-miR-US25-2-3p, which is known to target tissue inhibitors of metalloprotease 3 (TIMP3), resulting in an increased shedding of major histocompatibility complex class-I related chain A (MICA) and, again, decreasing NK cells recognition [85]. Additionally, HCMV miRNA UL148D (hcmv-miR-UL148D) also contributes to immune evasion by targeting the 3′UTR of the chemokine (C-C Motif) ligand 5 (CCL5) gene [86]. The CCL5 protein is a chemokine that induces NK cells proliferation and activation [87]; thus, hcmv-miR-UL148D, together with hcmv-miR-UL112 and hcmv-miR-UL112-1, contributes to HCMV NK evasion. Another mechanism of immune evasion was provided by a recent finding suggesting that hcmv-miR-UL112-3p targets toll-like receptors 2 (TLR2), inhibiting NFκB signaling and avoiding the associated inflammatory response [88]. Finally, the viral miRNA, hcmv-miR-US4-1, was confirmed to contribute to immune evasion by affecting antigen presentation by the major histocompatibility complex, class I (MHC-I) to cytotoxic T lymphocytes. The viral miRNA accomplishes this by targeting the endoplasmic reticulum aminopeptidase 1 (ERAP1) gene, preventing antigen peptide production [89]. Altogether, these viral miRNAs represent a potent viral mechanism of immune evasion by HCMV, acting together with other viral factors [90].

Another important group of cellular genes targeted by HCMV miRNAs are those associated with cell cycle control. By interfering with cell cycle control, HCMV is able to regulate the latent and lytic cycles, thus contributing to lifelong infection of the host. One important viral miRNA interfering with cell cycle is hcmv-miR-US25-1. This viral miRNA was found to target 20 different cellular transcripts. Among its targets were cyclin E2 (CCNE2), the collagenase stimulatory factor (CD147), the BRCA1/BRCA2-containing complex, subunit 3 (BRCC3), the EP300 interacting inhibitor of differentiation 1 (EID1), microtubule-associated proteins, the RP/EB family, member 2 (MAPRE2), and histone proteins (H3F3B), all with known functions in cell cycle control [91,92]. Interestingly, several hcmv-miR-US25-1 targets contained seed sequences in the 5′UTR of mRNA instead of the canonical 3′UTR [4], but the functional meaning of this finding has not yet been revealed [92]. Another cellular factor targeted by HCMV miRNAs is the eukaryotic initiation factor 4A1 (eIF4A1), an RNA helicase essential for translation initiation. Expression levels of eIF4A1 decreased due to hcmv-miR-US25-2-3p targeting [93]. This mechanism of translation inhibition by HCMV stands as a possible way to promote latency and cell cycle control.

In addition to immune evasion and cell cycle control, HCMV also targeted a group of cellular transcripts involved in vesicle trafficking. For example, the ATPase, H^+^ transporting, lysosomal 16 kDa, V0 subunit C pseudogene 1 (ATP6V0CP1) protein, which is required for acidification of endosomal compartments [94], is targeted by hcmv-miR-US25-1 viral miRNA. Similarly to other hcmv-miR-US25-1 targets, ATP6V0CP1 interference did not follow the canonical miRNA mechanism. Instead, viral miRNA seed sequences were located in the cellular gene open reading frame (ORF) [94], revealing yet another mechanism of interference for this particular viral miRNA. Interestingly, ATP6V0CP1 was found to be essential for viral replication, although the mechanism by which the absence of this cellular factor affects HCMV replication was not elucidated [94]. The fact that this cellular protein limits viral replication suggests that ATP6V0CP1 regulation may be important for viral control of replication and immune evasion during latency [94]. Three other viral miRNAs were found to target several genes of the secretory pathway. When collectively expressed, hcmv-miR-UL112-1, hcmv-miR-US5-1, and hcmv-miR-US5-2 targeted several proteins of this host pathway, including VAMP3 (vesicle-associated membrane protein 3), RAB5C (RAS-related protein 5C), RAB11A (RAS-related protein 11A), SNAP23 (synaptosomal-associated protein, 23 kDa), and CDC42 (cell division control protein 42) [95]. Interference of these host factors by HCMV-encoded miRNAs revealed another set of important functions inhibited by HCMV. This triple targeting affected the secretion of interleukin-6 (IL-6) and tumor necrosis factor α (TNF-α), the accumulation of transferrin in the endocytic recycling compartment (ERC), and the formation of the virion assembly compartment (VAC) [95]. Taken together, these effects of viral miRNAs were demonstrated to be essential for proper virion assembly [95].

### 4.2. Viral miRNAs Target HCMV Transcripts

Despite the fact that most identified HCMV miRNAs target cellular mRNAs, viral miRNAs can also interfere with HCMV transcripts. hcmv-miR-UL112-1 was identified to target a major immediate early trans-activator: the IE72. This immediate early viral transcript synthesis is important for HCMV DNA replication and expression of hcmv-miR112-1 reduced viral replication [91]. Interference with immediate early trans-activators is also observed with other herpesvirus-coded miRNAs, suggesting that targeting immediate early viral transcripts is an important mechanism to establish and maintain latency [96]. Interestingly, hcmv-miR-UL112-1 is encoded antisense to viral uracil DNA glycosylase (UL114), which increases both early and late viral transcripts synthesis [97]. Again, this miRNA poses as a viral factor that favors latency. Another viral gene targeted by viral miRNAs is the US7 protein. Although the functions of this viral gene remain elusive, the mechanism by which viral miRNAs target US7 3′UTR demonstrated a synergic repression by two viral miRNAs. In fact, hcmv-miRUS5-1 and hcmv-miRUS5-2 are encoded antisense to US7 3′UTR, and this finding stood as the first evidence of two HCMV miRNAs acting cooperatively [98]. Finally, hcmv-miR-UL36 was thought to target UL138, a determinant gene for latent infection [99], but the relevance of this miRNA remains uncertain, as it was not detected among latency-associated miRNAs [100].

### 4.3. HCMV-Encoded miRNAs Are Expressed Differentially in Latent and Lytic Infection

Similar to studies conducted with other viruses, HCMV miRNA expression changes between latent and lytic infection. In a recent report, eight viral miRNAs were found in latently infected cells, with hcmv-miR-UL112-3p and hcmv-miR-US22-5p being the two most abundant miRNAs during latency [100]. Interestingly, the majority of these miRNAs are encoded in the unique long (UL) region of the viral genome, suggesting the existence of regulatory mechanisms important for transition to the latent phase. In addition, a subset of viral miRNAs highly expressed in lytic infection (hcmv-miR-US25-2-5p, hcmv-miR-US29-5p, hcmv-miR-US25-1-5p, and hcmv-miR-US25-2-5p) presented a significant decay during the course of infection and establishment of latency [100]. Simultaneously, five miRNAs, including hcmv-miR-UL112-3p and hcmv-miR-US22-5p, were continuously expressed throughout infection by HCMV [100]. Interestingly, three miRNAs (*i.e.*, hcmv-miR-US25-2-5p, hcmv-miR-US25-1-5p, and hcmv-miR-UL112-3p) show remarkable increase upon reactivation of lytic infection, demonstrating a distinct pattern of viral miRNA expression in lytic and latent infection [100]. Another intriguing finding was that hcmv-miR-US29 differentially expressed two mature miRNAs from two strands of pre-miRNA. Hence, during lytic infection, hcmv-miR-US29-5p was expressed and was undetectable during latent phase, while the opposite occurred with hcmv-miR-US29-3p [100]. This was the first demonstration of a viral mechanism that apparently is related to transition to latency.

### 4.4. HCMV Transcripts Alter Cellular miRNA Expression

HCMV infection causes profound changes in cellular miRNAs. Although the consequences of these changings are not completely understood, it is conceivable that HCMV alters cellular miRNA expression to favor viral replication. In a recent study, 49 cellular miRNAs were expressed differentially during HCMV infection [101]. Among those, 39 were upregulated and 10 were downregulated during latent infection. Unsurprisingly, several cellular miRNAs known to have antiviral functions were downregulated. Interestingly, besides its antiviral activity, downregulated hsa-miR-21 was also reported to have an important role in birth defects caused by congenital HCMV infection [102,103], contributing to the neuropathogenesis of HCMV infection of fetal central nervous system. On the other hand, hsa-miR-124, known to modulate monocyte and macrophage activation, was upregulated during HCMV latent infection. By inhibiting macrophage activation, hsa-miR-124 helps to create a cellular environment suitable for HCMV latent infection [101]. Moreover, cellular miRNAs deregulated by HCMV are thought to be linked to several important cellular functions such as cell proliferation, differentiation, and oncogenesis [104]. Together, these findings point to an interesting possibility to develop miRNA-based-therapies to reduce HCMV infection severity.

### 4.5. Cellular miRNAs Target Viral Transcripts and Promote HCMV Latency

Despite the described cellular miRNA deregulation by HCMV, one family of cellular miRNAs was discovered to target a viral immediate early transcript. UL112 is targeted by three of the five hsa-miR-200 miRNA family members. Hsa-miR-200b, hsa-miR-200c, and hsa-miR-429 target the 3′UTR of UL112 [105]. This immediate early transcript is one of two immediate early proteins that are known to start HCMV reactivation. Moreover, the hsa-miR-200 cluster also appears to be highly expressed in undifferentiated cells such as monocytes. Therefore, the presence of high levels of hsa-miR-200 miRNA in undifferentiated cells may promote latency by suppressing a key protein for HCMV gene expression, and the loss of this miRNA during differentiation may stand as an important switch that promotes lytic infection [105].

In conclusion, HCMV infection is a complex and dynamic process where cellular and viral factors interact, resulting in lifelong infection by the virus. miRNAs play an important role in HCMV infection, and their functions are still being deciphered. In the future, HCMV miRNAs may provide important information about HCMV infection, namely as diagnostic and prognostic markers of disease outcome [106]. Additionally, miRNAs can provide new therapeutic targets and novel approaches to HCMV treatment, similar to what happens with other viruses [107,108].

## 5. Gammaherpesviruses: Epstein–Barr Virus (EBV)-Encoded miRNAs

**Figure 3:**
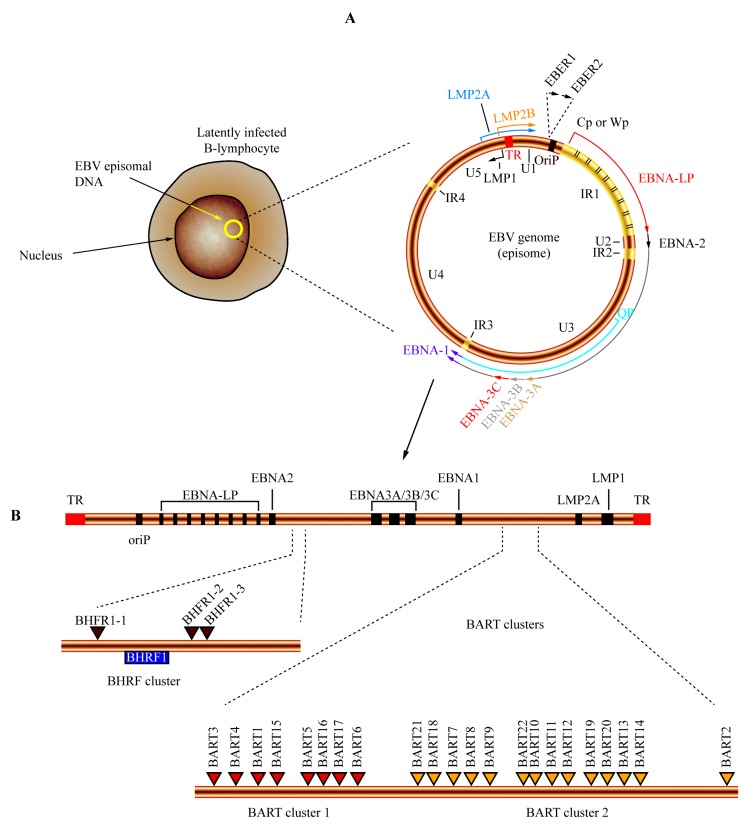
Genome organization and location of miRNAs encoded by the Epstein–Barr virus (EBV). (**A**) After primary infection, EBV establishes a latent infection in B-lymphocytes. During latency, the EBV genome is maintained as a circular episome (represented as a yellow circle inside the nucleus). The general structure of episomal DNA is presented, and the relative positions of the latency-associated genes on the viral episome are shown. The origin of plasmid replication (oriP) is shown in brown. The outer circle represents the coding regions of the latent proteins transcribed in antisense (LMP1, black arrow) and sense orientation (EBNA-LP, red; EBNA2, black; EBNA3A, light brown; EBNA3B, light grey; EBNA3C, red; EBNA1, purple; LMP2A, blue; LMP2B, orange). EBNA-LP is transcribed from variable numbers of repetitive exons (denoted by black lines inside the IR1 region). The highly transcribed non-polyadenylated RNAs EBER1 and EBER2 are represented in the top of the diagram. The outer long circular arrow represents the EBV transcript during the latency III program where all the EBNA genes are transcribed from Cp or Wp promoters. The inner blue arrow line represents the EBNA1 transcript originated from the Qp promoter during latency I and latency II programs. U1-U5 refers to largely unique DNA domains while IR1-IR4 represents internal repetitive DNA domains. TR (represented in red) denotes the terminal repeat of EBV DNA; (**B**) Genomic location of miRNAs encoded by EBV. A linear representation of EBV genome is shown and the positions of the main latent genes are represented by black boxes; a dark brown box denotes oriP position. The regions of the BHRF cluster and BART clusters 1 and 2 are expanded in the bottom of the diagram. The locations of miRNA precursors are denoted by brown (BHRF cluster), red (BART cluster 1) and yellow (BART cluster 2) triangles.

The Epstein–Barr virus (EBV) is a herpesvirus that, similarly to other herpesviruses, establishes a lifelong latent infection. The most common manifestation of EBV primary infection is a clinical syndrome known as infectious mononucleosis, usually affecting adolescents and young adults. After initial infection, EBV genomic DNA is maintained as episomes in the nucleus of memory B-lymphocytes. [109]. Occasionally, the virus is reactivated and infects new cells (e.g., B-lymphocytes and epithelial cells), and infectious viral particles are shed mainly through saliva. Although EBV infection is usually benign, the most relevant aspect of EBV pathology is the ability to transform infected cells [109]. Given its transforming capacity, EBV has been associated with several cancer types. EBV is found in almost all cases of undifferentiated nasopharyngeal carcinoma (NPC), nasal NK/T-cell lymphoma (NKTL), and post-transplant lymphoma (PTLD), in sporadic and endemic Burkitt’s lymphoma (BL) (15%–30% and 95% of the cases, respectively), in some subsets of Hodgkin’s disease (HD), in diffuse large B-cell lymphoma (DLBCL) (15% of the cases), and in gastric carcinoma (GC) (10% of the cases) [110]. Interestingly, EBV expresses different latency-associated genes depending on the infected cell, giving rise to four different patterns of gene expression known as latency types 0, I, II, and III [111]. Different latency types are found in different EBV-associated diseases [111]. Therefore, it is no surprise that EBV-encoded miRNAs are also differentially expressed depending on the infected cell and the host immune control [112]. EBV encodes 44 mature miRNAs from 25 precursors [113,114,115,116,117,118,119] (Figure 3). Viral miRNAs are distributed by three clusters: BHRF1-cluster, BART-cluster 1, and BART-cluster 2 [34].

### 5.1. EBV-Encoded miRNAs Help Immune Evasion

EBV miRNAs target both cellular and viral mRNAs to evade the immune system (Table 1). Viral miRNAs are able to supress viral antigens LMP1 and LMP2A, two latency-associated membrane proteins. Three different EBV miRNAs from BART-cluster 1 (ebv-miR-BART16, ebv-miR-BART17-5p, and evb-miR-BART1-5p) co-target LMP1 [120]. Despite being a known antigen, this modulation of LMP1 was not shown to be directly correlated with a decreased immune response. However, due to its role as pro-apoptotic protein, it is expected that the downmodulation of LMP1 provides an indirect mechanism of immune evasion [37,120]. Concerning the other latency-associated membrane protein, LMP2A, a similar mechanism for immune evasion is proposed, pointing to a modulation of this viral protein resulting in immune surveillance escape [117]. This was shown due to high sequence complementarity between LMP2 and ebv-miR-BART22. Furthermore, high levels of the viral miRNA correlated with low levels of this viral transcript [117], providing strong evidence that this viral miRNA targets LMP2A. As observed with many other miRNAs, it should be emphasized that some of these functions, although suggesting potential mechanisms of immune evasion, are in general not completely demonstrated.

Alternatively, viral miRNAs can directly target cellular mRNAs involved in host immune response. Viral miRNA ebv-miR-BHRF1-3 targets CXCL11, a T-cell attracting chemokine [121], thus contributing to immune evasion by modulating host’s mechanisms of defence. Similar to HCMV, EBV miRNAs can also interfere with MICB. In fact, ebv-miR-BART2-5p has been shown to target this NK cell ligand, hence reducing cell recognition and again contributing for immune evasion [124]. Finally, ebv-miR-BART15 was found to bind NLRP3 in the same site as hsa-miR-223 [130]. NLRP3 (NLR family, pyrin domain containing 3; also known as cryopyrin) is a cellular protein that contributes to the production of pro-inflammatory cytokines such as IL-1β and IL-18. Thus, by targeting this cellular transcript, EBV reduces immune response, providing an additional mechanism to evade immune response through the expression of its miRNAs.

### 5.2. EBV miRNAs Avoid Apoptosis by Targeting Cellular Pro-Apoptotic Genes

Viral miRNAs play a key role in avoiding cell death, mostly by inhibiting the expression of cellular pro-apoptotic genes [135] (Table 1). One of the best characterized cellular targets of EBV miRNAs is the pro-apoptotic protein PUMA (p53-upregulated modulator of apoptosis) [126]. PUMA is regulated by ebv-miR-BART5-5p, and depletion of this viral miRNA or induction of PUMA expression is sufficient to trigger apoptosis [126]. Another pro-apoptotic protein modulated by EBV is BIM (BCL2 interacting mediator of cell death), a member of BH3-only family proteins. Several EBV-encoded miRNAs from BART-cluster 1 are involved in this regulation, especially ebv-miR-BART1, ebv-miR-BART3, ebv-miR-BART9, ebv-miR-BART11, and ebv-miR-BART12 [122]. Many other cellular pro-apoptotic proteins seem to be modulated by EBV’s miRNAs. For example, TOMM22 (translocase of outer mitochondrial membrane 22 homolog), part of the mitochondrial pore receptor complex for the pro-apoptotic protein BAX (BCL2-associated X protein), was identified as a potential target for ebv-miR-BART16 [122]; the BCL2-associated death promoter (BAD) protein, also from the BH3-only pro-apoptotic proteins, is targeted by the viral miRNA ebv-miR-BART20-5p [131]; finally, the pro-apoptotic protein caspase 3 was identified as being a target of ebv-miR-BART16 and ebv-miR-BART1-3p [123]. Together, these findings suggest an important role for EBV miRNAs, avoiding apoptosis both at early stages of infection but also upon cell transformation.

### 5.3. EBV-Encoded miRNA ebv-miR-BART6-5p Targets Dicer

One viral miRNA, ebv-miR-BART6-5p, was found to target Dicer mRNA, reducing the biogenesis of cellular miRNAs [127,128] (Table 1). This Dicer interference by a viral miRNA probably results in a negative feedback loop that can act as a tight control for viral and cellular miRNAs [128]. Interestingly, downregulation of Dicer can also serve another purpose, as it has been suggested that Dicer plays a role in EBV reactivation [132] Moreover, ebv-miR-BART6-5p was found to undergo RNA editing that decreases ebv-miR-BART6-5p loading into RISC [128]. This post-transcription editing of pri-miRNA stands as a possible cellular mechanism of defence against viral infection that EBV counteracts by mutating pri-miRNA sequence [128].

### 5.4. Cellular miRNAs Regulate EBV Switch from Latent to Lytic Infection

Several cell-encoded miRNAs modulate the latent/lytic cycles during EBV infection (Table 1). EBV reactivation starts with the expression of BZLF1 viral transcript. This viral transcript is supressed by ZEB1 and ZEB2 host proteins that bind BZLF1 promoter [136,137]. ZEB1 and ZEB2 are targeted by cellular miRNAs from the hsa-miR-200 family. Expression of hsa-miR-200b and hsa-miR-429 has been shown to induce EBV reactivation upon regulation of ZEB proteins [133,134]. Another mechanism modulating EBV reactivation involves the cellular miRNA hsa-miR-let-7a. Apparently, viral protein EBNA1 upregulates hsa-miR-let-7a, which in turn decreases the level of cellular protein Dicer (the target of hsa-miR-let-7a). Since Dicer contributes to EBV reactivation, the induction of hsa-miR-let-7a by EBNA1 promotes EBV latency [132]. Therefore, together with ebv-miR-BART6, hsa-miR-let-7a plays an important role downregulating Dicer, contributing to EBV latency probably by decreasing cellular miRNA levels that can reactivate EBV [127,128,132].

### 5.5. EBV miRNAs Target Tumor Suppressor Genes

Besides cellular proteins related with immune response or apoptotic pathway, some EBV miRNAs were shown to target other cellular mRNAs important for normal cell function. Among these are several tumor-suppressor genes and a key protein of miRNA pathway (Table 1). Viral miRNA interference of tumor-suppressor proteins expression facilitates typical B-cell transformation into indefinitely proliferating lymphoblastoid cell lines as observed upon EBV infection. For example, the EBV-encoded miRNA, ebv-miR-BART3-5p, targets DICE1 (deleted in cancer 1), a tumor-suppressor gene; consequently, the expression of ebv-miR-BART3-5p *in vitro* resulted in the proliferation of host cells [125]. EBV miRNAs also target two other tumor-suppressor genes: ebv-miR-BART19-3p targets WIF1 (WNT inhibitory factor 1), while ebv-miR-BART7 and ebv-miR-BART19-3p target APC (adenomatous polyposis coli) [129]. Additionally, EBV was demonstrated to induce the expression of the oncomir hsa-miR-155 [138,139]. Expression of this well-known cellular miRNA was demonstrated to be crucial for B-cell transformation and proliferation [140]. This complex network of interactions between EBV-encoded miRNAs and their targeted genes provides crucial information on EBV-associated tumorigenesis, as detailed in the following section.

### 5.6. EBV miRNAs Play an Important Role in Viral-Induced Carcinomas and Lymphomas

Several EBV-associated cancers were studied regarding both viral and cellular miRNAs expression profiles. These studies revealed a high inter-tumor variation of viral miRNA expression and posed some challenges, particularly deriving from cellular infiltrates that occur in different tumors and to diverse degrees.

One of the most prominent EBV-associated tumors is NPC. Comparison of the NPC tissue miRNA profile *vs.* non-tumor tissue revealed that 5%–19% of miRNAs expressed in the tumor are EBV-encoded [119]. Although no viral miRNA was found to be consistently expressed in NPC, ebv-miR-BART7-3P, ebv-miR-BART22, and miRNAs from BART-cluster 1 were described as being highly expressed in this kind of tumor [114,119,141]. Conversely and despite the inter-tumor variability, all reports indicate the absence of miRNAs from BHRF1-cluster. In addition, several cellular miRNAs were found to be either up- or downregulated in NPC. Among those upregulated, we may mention hsa-miR-17-5p, hsa-miR-320, hsa-miR-652, while the downregulated miRNAs were hsa-miR-15a, hsa-miR-16, hsa-miR-23a/b, and hsa-miR-200c [119]. Since there are several possible targets for these deregulated cellular miRNAs, it is difficult to anticipate the outcome of their decreased or increased expression. It will be interesting to find whether this deregulation of cellular miRNAs is due to EBV infection or if it is a consequence of cellular transformation. Furthermore, it will be important to elucidate these cellular miRNA functions in NPC.

Similarly to NPC, studies concerning GC revealed the predominance of viral miRNAs from BART-clusters and an almost total absence of BHRF1-cluster miRNAs [142,143]. Moreover, several cellular miRNAs were found to be downregulated in GC. Members of the let-7 family and hsa-miR-200 were among the most downregulated host miRNAs. Interestingly, several downregulated cellular miRNAs were tumor-suppressor miRNAs [143]. Taken together, these findings confirm the role of both cellular and viral miRNAs in the well-defined oncogenic potential of EBV.

Alongside NPC and GC, EBV-associated lymphomas were also studied regarding both viral and cellular miRNAs expressions. In DLBCL, viral miRNAs from BART-cluster 2 were slightly more expressed than other viral miRNAs [144]. In contrast, the viral miRNAs ebv-miR-BART7, ebv-miR-BART22, and ebv-miR-BART10 were highly expressed in cells from DLBCL, while ebv-miR-BART15 and ebv-miR-BART20, along with miRNAs derived from BHRF1-cluster, were not detected [144]. Similar to NPC and GC, numerous cellular miRNAs were differentially expressed in EBV-positive and EBV-negative DLBCL. The cellular miRNAs upregulated in EBV-positive DLBCL were hsa-miR-424, hsa-miR-223, hsa-miR-199a, hsa-miR-27b, hsa-miR-378, hsa-miR-26b, and hsa-miR-23a/b. Conversely, miRNAs downregulated were hsa-miR-20b, hsa-miR-221, hsa-miR-151-3p, hsa-miR-222, hsa-miR-29b/c, and hsa-miR-106a. For most of these miRNAs, a role in cancer pathogenesis has been described [12,15], which thereby sustains their role in EBV oncogenesis. Interestingly, Acquired Immunodeficiency Syndrome (AIDS)-related DLBCL cells express high levels of ebv-miR-BART22 and ebv-miR-BHRF1-3, in opposite to cells from immunocompetent patients with DLBCL, suggesting that these miRNAs may play a role in regulation of host cell immune response [121].

Likewise, AIDS-related BL presented a manifest difference regarding miRNA expression depending on EBV latency type. Similar to other EBV type I latency lymphomas and carcinomas, AIDS-related BL did not express miRNAs from BHRF1-cluster, whereas, in the type III latency BL, miRNAs from BHRF1-cluster were abundantly expressed [121]. Interestingly, in both AIDS-related lymphomas (DLBCL and BL), BHRF1-cluster expression was accompanied by the detection of LMP1 [121]. Moreover, BHRF1-miRNAs expression is dependent on latency III promoters, and BHRF1 transcripts are induced in lytic cycle activation [145]. Therefore, these data suggest a different behavior of EBV-related lymphomas in immunocompromised patients probably because EBV does not have the need to regulate its gene expression so strongly given the lack of an effective immune response to viral transcripts.

In NKTL, viral miRNAs expressed were similar to other lymphomas: BHRF1-cluster miRNAs were not detected, whereas ebv-miR-BART5 and ebv-miR-BART7-5p were the most highly expressed viral miRNAs [146]. In addition, a group of 15 upregulated and 16 downregulated cellular miRNAs were detected. Among downregulated cellular miRNAs were hsa-miR-142-3p, which targets the pro-inflammatory IL-1α, and hsa-miR-205, which targets the oncogenic protein BCL6 [146].

Recent studies suggest that EBV miRNAs can also play an important role in cancer progression. For instance, ebv-miR-BART-7-3p, highly regulated in NPC, was shown to target tumor-suppressor phosphatase and tensin homolog (PTEN), modulating PI3K/Akt/GSK-3β signaling, resulting in epithelial to mesenchymal transition and favoring metastasis [147]. A similar mechanism was suggested for ebv-miR-BART1, which also targets PTEN [148]. These two findings reinforce the idea that viral miRNAs are redundant and sometimes share cellular targets to ensure its repression. In addition to these findings, all viral miRNAs from BART-cluster 2 were also demonstrated to target N-myc downstream regulated 1 (NDRG1) cellular transcript [149]. NDRG1 encodes a suppressor of metastasis and an epithelial differentiation marker, and its suppression may avoid cell growth arrest and terminal differentiation, providing EBV with a new mechanism for EBV-mediated carcinogenesis [149]. Taken together, all these findings demonstrate that EBV miRNAs are important to explain viral oncogenic properties.

### 5.7. EBV Is Able to Transfer Viral miRNAs through Exosomes

EBV infection of target cells can induce the transfer of miRNAs and proteins via exosomes [150,151]. The release of these exosomes was observed in cells from NPC [152], and they transported miRNAs from all different clusters as well as LMP1 protein [151]. Viral miRNAs were capable of targeting mRNAs in neighbor cells and probably even more distant cells, as these exosomes were detectable in serum samples [152]. These findings may help to explain the occurrence of multiple sclerosis and its association with EBV. In fact, almost all cases of multiple sclerosis are found in EBV-positive patients. However, the absence of viral DNA in inflammatory lesions caused by this disease has challenged the link between EBV and multiple sclerosis; even so, it is possible that viral miRNAs carried by exosomes provide a mechanism that could explain the role of EBV in multiple sclerosis etiology [153,154,155,156,157].

In conclusion, the complex interactions between EBV miRNAs, host proteins, and host miRNAs contribute largely for viral latency and help the onset of lifelong infection of EBV, similarly to other herpesvirus. Moreover, EBV miRNAs play an important role in immune evasion, cell transformation, and proliferation. Therefore, viral miRNAs contribute, together with several other viral proteins, to oncogenic processes and help to explain EBV-associated B-cell lymphomas as well as carcinomas. Interestingly, miRNAs carried by exosomes can be the missing link between EBV and multiple sclerosis.

## 6. Human Herpesvirus 8/Kaposi’s Sarcoma-Associated Herpes Virus (HHV-8/KSHV) Encodes 25 Mature miRNAs

**Figure 4:**
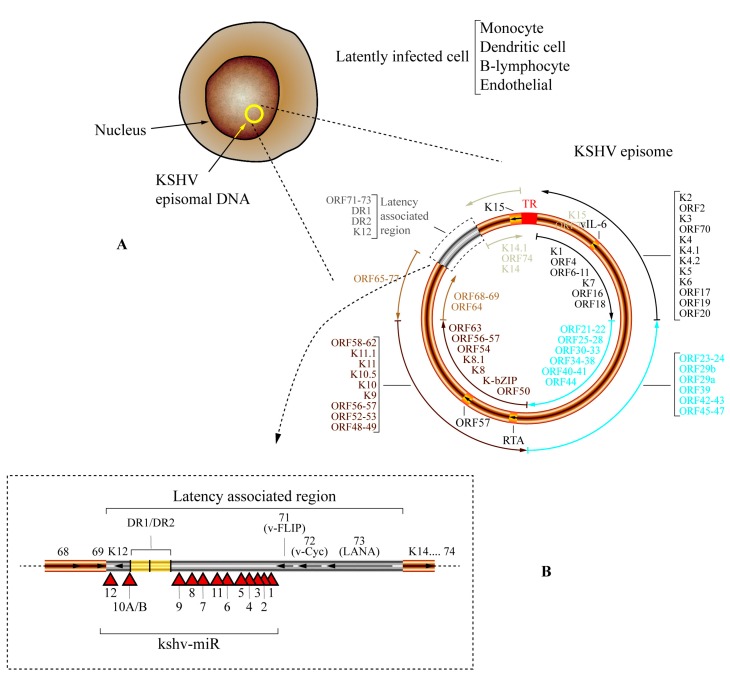
Genome organization and location of miRNA encoded by Kaposi’s sarcoma-associated herpesvirus (KSHV) (HHV-8). (**A**) After primary infection, KSHV establishes a latent infection in monocytes, dendritic cells, B-lymphocytes, and endothelial cells. During latency, the KSHV genome is maintained as a circular episome (represented as a yellow circle inside the nucleus). The general structure of episomal DNA is presented, and the relative positions of the open reading frames (ORFs) are shown. The grey box represents the latency-associated region. The terminal repeat (TR) region is represented by a red box, and the ORFs encoding for viral interleukin 6 (vIL-6), the replication and transcription activator (RTA), ORF57, and K15 are represented by light orange boxes. The outer circular arrows refer to the ORFs that are transcribed in antisense orientation, while the inner circular arrows refer to those that are transcribed in the opposite orientation. To facilitate the representation of the different ORFs and their relative location, and the outer and inner circular arrows are presented in different colors (black, light blue, brown, light brown and light grey); (**B**) Genomic location of miRNAs encoded by KSHV (kshv-miR). A linear representation of the latency-associated region (grey box) of the KSHV genome is shown, and the locations of miRNA precursors are denoted by red triangles. The positions of ORFs 68, 69, K12, 71 (v-FLIP), 72 (v-Cyc), 73 (LANA), K14, and 74 are also shown; arrows inside each ORF indicate the direction of transcription. The two adjacent yellow boxes represent the DR1/DR2 region.

Human herpesvirus 8 (HHV-8)—also known as Kaposi’s sarcoma-associated herpes virus (KSHV)—is a herpesvirus from the *Gammaherpesvirinae* subfamily. KSHV is known as the etiologic agent of several cancers, such as Kaposi’s sarcoma (KS), primary effusion lymphoma (PEL), and multicentric Castleman’s disease (MCD), particularly in immunosuppressed patients (e.g., HIV-infected patients and solid organ transplant recipients undergoing immunosuppressive treatment) [39,158]. Like all herpesviruses, KSHV establishes a latent infection in various cell types, namely in monocytes, dendritic cells, B-lymphocytes, and endothelial cells. KSHV encodes 13 pre-miRNAs, originating 25 mature miRNA sequences. These 13 pre-miRNAs are unevenly distributed throughout the KSHV genome (Figure 4). kshv-miR-K12-1 to kshv-miR-K12-9 and kshv-miR-K12-11 are encoded within K12 intron, while kshv-miR-K12-10a,b is located within the K12 ORF, and kshv-miR-K12-12 is transcribed within the 3′UTR of K12 [78,159,160]. Expression levels of KSHV miRNAs vary considerably [159], and, while some are highly conserved (e.g., kshv-miR-K12-1, kshv-miR-K12-3, kshv-miR-K12-8, kshv-miR-K12-10, kshv-miR-K12-11, and kshv-miR-K12-12), others exhibit alterations that may affect the respective processing and function [161,162]. Moreover, these polymorphisms may correlate with KS risk [163].

KSHV miRNAs seem to play a role in viral pathogenesis in a disease-specific manner and it is possible that viral miRNAs exhibit different functions depending on the infected cell’s type [39]. KSHV miRNAs target multiple cellular genes important to normal cell functioning. Furthermore, KSHV miRNAs contribute to immune evasion and modulation, avoid apoptosis, and contribute to tumorigenesis (Table 2). Besides miRNAs, other viral proteins interfere with cellular miRNAs and their functions.

### 6.1. KSHV miRNAs Target Cellular mRNAs to Evade Immune Response and Modulate Cytokines Response

KSHV-encoded miRNAs can inhibit innate immune response. kshv-miR-K12-9 targets IRAK1 (interleukin-1 receptor-associated kinase 1) while kshv-miR-K12-5 targets MYD88 (myeloid differentiation primary response 88). Both IRAK1 and MYD88 mediate TLR/IL-1R signaling, and their repression results in reduced inflammation [175]. Furthermore, kshv-miR-K12-11 targets IκB kinase epsilon (IKKε), resulting in attenuated type I interferon signaling [179]. Similar to HCMV and EBV, KSHV evades NK cell recognition and killing by encoding a miRNA, kshv-miR-K12-1, that targets MICB mRNA [124].

In addition to the modulation of NK cell recognition, KSHV also modulates cytokine secretion. One viral miRNA, kshv-miR-K12-10, targets the tumor necrosis factor-like weak inducer of the apoptosis receptor (TWEAKR). Repression of TWEAKR reduces interactions with its ligand, resulting in reduced expression of IL-8 and monocyte chemoattractant protein 1 (MCP-1) [178]. Moreover, kshv-mir-K12-3 and kshv-miR-K12-7 repress C/EBPβ (CCAAT/enhancer-binding protein β), a transcriptional repressor of IL-6 and IL-10 [168], two cytokines that promote cell growth of KSHV-infected cells, and angiogenesis [158]. However, it remains unclear if these two viral miRNAs target C/EBPβ directly or indirectly by targeting other upstream factors [158]. In contrast, kshv-miR-K12-11 directly targets C/EBPβ and has been shown to contribute to splenic B-cell expansion and KSHV-associated lymphomagenesis [180]. Therefore, it is well established that KSHV miRNAs cooperatively regulate cytokine expression to facilitate infection and oncogenesis while avoiding the host immune system.

### 6.2. KSHV-Encoded miRNAs Regulate Cell Growth and Survival

KSHV is a well-known oncogenic virus. Therefore, its ability to avoid cell death and promote cell cycle progression and cell growth is closely related with KSHV-induced cell transformation. KSHV contributes to these functions by targeting key cellular proteins involved in these pathways. For example, kshv-miR-K12-1 targets the cellular cyclin-dependent kinase inhibitor p21 to promote cell cycle progression [164]. The same viral miRNA was also shown to interfere with IκBα, activating the NFκB pathway [165], which contributes to cell survival. Similarly, kshv-miR-K12-10 targets TWEAKR, conferring resistance to TWEAK-induced apoptosis, which is normally mediated by TWEAK/TWEAKR interactions [178]. In addition, this viral miRNA also interferes with transforming growth factor beta (TGF-β) receptor II (TGFBR2), affecting the TGF-β pathway [166]. Interestingly, the same pathway is also targeted by another viral miRNA, kshv-miR-K12-11, which targets SMAD5 (SMAD family member 5), an intermediate in the TGF-β pathway [181]. This inhibition of the TGF-β pathway ultimately results in increased cell survival and virally induced oncogenesis [166,181].

Viral-driven cell transformation and oncogenesis relies heavily on the ability to avoid apoptosis. Therefore, unsurprisingly, three other viral miRNAs, kshv-miR-K12-1, kshv-miR-K12-3, and kshv-miR-K12-4-3p, cooperatively suppress caspase 3 by binding its 3′UTR and reducing apoptosis in infected cells [167]. Furthermore, several viral miRNAs regulate cellular transcription-factors that indirectly protect infected cells from reactive nitrogen and oxygen species-induced apoptosis [168,169].

An obvious conclusion is that KSHV-encoded miRNAs target multiple pathways related to cell survival. Interestingly, some viral miRNAs target multiple pathways, and the same pathway can be targeted by multiple viral miRNAs. This evidences the redundancy of viral miRNA functions and the importance of KSHV miRNAs in cell transformation and oncogenesis. In fact, some studies have shown that KSHV mutants lacking 10 viral miRNAs encoded in K12 intron failed to transform infected cells and ultimately induced cell apoptosis [182].

### 6.3. KSHV miRNAs Facilitate Virus Entry

Several miRNAs were associated with increased susceptibility to KSHV infection. Among them, kshv-miR-K12-1, kshv-miR-K12-9, and kshv-miR-K12-11 were shown to increase xCT expression, a fusion-entry receptor for KSHV, increasing macrophage and endothelial cell susceptibility to KSHV [168]. Consistently, kshv-miR-K12-11 has been shown to target BACH-1 (BTB and CNC homology 1) which, in turn, is a negative regulator of xCT [170,171]. Several viral miRNAs—in particular kshv-miR-K12-11 and kshv-miR-K12-6—cooperatively downregulate a cellular transcription-factor MAF (v-maf avian musculoaponeurotic fibrosarcoma oncogene homolog) [169]. MAF also negatively regulates xCT, underlining the cooperative and redundant action of viral miRNAs. These findings point to a KSHV evolutionary strategy that promotes infection by increasing cell susceptibility to virus entry [158], a crucial step in viral replication cycle.

### 6.4. KSHV miRNAs Target Viral Transcripts to Regulate Latent and Lytic Infection

Similar to other herpesviruses included in this review, KSHV miRNAs also target viral and cellular mRNAs to promote latency. kshv-miR-K12-7 and kshv-miR-K12-9 target immediate-early gene ORF50, decreasing the expression of the replication and transcription activator (RTA) encoded within this viral gene [176,177] (Figure 4). Additionally, kshv-miR-K12-4 repress the retinoblastoma-like protein 2 (RBL2) that indirectly maintains methylation of the RTA promoter and consequently reduces expression of this viral gene [173]. Another viral miRNA that impairs RTA expression is kshv-miR-K12-3. This KSHV-encoded miRNA targets the nuclear factor I/B (NFIB), known to act as a RTA activator [172]. The RTA protein is essential for initiation of lytic replication of KSHV; therefore, interfering with this viral transcript promotes viral latency. Lastly, kshv-miR-K12-1 also contributes to latency by targeting IκBα, as previously mentioned, which leads to NFκB-dependent viral latency [166].

Conversely, viral miRNAs can also induce lytic replication cycle, essential for spreading the infection. Targeting of BCLAF1 (BCL2-associated transcription factor 1) by kshv-miR-K12-5 and kshv-miR-K12-9 promotes lytic replication through unknown mechanisms [174]. Moreover, kshv-miR-K12-11 repression of IKKε also contributes to lytic reactivation of KSHV [179]. In addition, chemical induction of KSHV reactivation revealed upregulation of kshv-miR-K12-10 and kshv-miR-k12-12, suggesting that these viral miRNAs may also play a role in KSHV lytic reactivation [162].

Taken together, these findings evidence the importance of viral miRNAs in the regulation of KSHV lytic and latent infection. Apparently, viral miRNAs predominantly promote latent infection. This observation is in agreement with miRNAs’ location within the KSHV genome: all of them are encoded within the latency-associated region [159].

### 6.5. KSHV Encodes Orthologues of Cellular miRNAs

Three viral miRNAs share perfect seed homology with cellular miRNAs [170,171,183,184]. For instance, kshv-miR-K12-10 is a viral orthologue of hsa-miR-142-3p. Interestingly, kshv-miR-K12-10 can be processed into four different functional variants. In PEL cells, hsa-miR-142-3p presented a very similar processing pattern, and hsa-miR-142-3p variants’ seed sequence matched those from kshv-miR-K12-10 variants [166,183]. Viral and cellular miRNAs were shown to target TGF-β type II receptor, inhibiting the TGF-β pathway [166]. In addition, kshv-miR-K12-11 shares seed sequence with hsa-miR-155 [170,171], a well-known oncomir [185]. Again, both miRNAs are known to target IKKε, C/EBPβ, BACH-1, and SMAD5 [168,179,180,181,185]. Lastly, kshv-miR-K12-3 was found to share seed sequence homology with hsa-miR-23. Moreover, cellular and viral miRNAs were shown to share targets, such as caspase 3 and caspase 7, linking these two miRNAs with anti-apoptotic functions [184]. By encoding orthologues, KSHV is able to ensure the correct expression of key miRNAs necessary for viral infection and persistence. Moreover, by sharing seed sequences with cellular miRNAs, these viral miRNAs can be directed to the same targets as host miRNAs but can also target other genes and *vice versa*, adding an increased layer of complexity to viral miRNA regulation of host genes.

### 6.6. Cellular miRNAs Play a Key Role in KSHV Pathology

Considering the many functions of the cellular miRNAs, it is no surprise that KSHV-mediated deregulation of these miRNAs can contribute to viral pathogenesis [186]. Deregulation of cellular miRNAs may promote KSHV-associated cancers, immune modulation, and regulation of the viral replication cycle [186].

A viral protein, K15 (Figure 4), has been shown to upregulate the expression of hsa-miR-21 and hsa-miR-31 [187]. These two miRNAs promote cell migration, angiogenesis, and lymphangiogenesis, indicating an important role in the development of cancer, especially in metastatic processes [187]. Remarkably, cellular miRNAs from the hsa-miR-221/hsa-miR-222 cluster were found to be greatly downregulated in KSHV-infected cells. Viral latency-associated nuclear antigen (LANA) and Kaposin B are thought to mediate the repression of these cellular miRNAs, which results in increased cell migration [188]. Moreover, KSHV was also found to downregulate hsa-miR-30b/c. These two cellular miRNAs target Delta-like 4 (DLL4), a protein involved in vascular development and angiogenesis [189]. Interestingly, all these miRNAs were previously identified as either oncogenes or tumor-suppressor genes in several types of cancer [186]. Thus, KSHV-mediated deregulation of this cellular miRNAs appears to play an important role in viral oncogenesis.

An important cellular chemokine receptor, CXCR4, is targeted by hsa-miR-146a. These cellular miRNAs is overexpressed in response to NFκB activation by viral FLICE inhibitory protein (vFLIP; Figure 4) [190]. Interestingly, repression of CXCR4 appears to contribute to KS by promoting the premature release of endothelial cell progenitors infected with KSHV into the blood stream [190]. In addition to promoting IL-6 expression by miRNA-mediated repression of C/EBPβ (mentioned earlier in this review), KSHV also encodes a viral interleukin 6 (vIL-6; Figure 4) that mimics its human cognate. IL-6 and vIL-6 are targeted by hsa-miR-608 and hsa-miR-1293, respectively [191]. Remarkably, the viral ORF57 protein (Figure 4) has been shown to compete with these two cellular miRNAs for the binding sites in IL-6 and vIL-6 mRNAs, avoiding RISC-mediated suppression of both mRNAs [192]. The expression pattern of cellular miRNAs upon KSHV infection revealed that hsa-miR-146a, hsa-miR-31, and hsa-miR-132 peaked at 6 hours post-infection, while hsa-miR-193a and hsa-miR-let-7i steadily increased over 72 h post-infection [193]. Moreover, hsa-miR-132, one of the upregulated cellular miRNAs, has been shown to repress interferon-stimulated genes by targeting p300 transcriptional co-activator [193].

There is little evidence that cellular miRNAs can regulate KSHV lytic or latent infection by targeting viral transcripts. However, studies that considered the influence of HIV-1 Nef protein on the KSHV replication provided interesting insights regarding the role that cellular miRNAs may play in KSHV latent and lytic infection [194]. For example, hsa-miR-1258 was confirmed to target RTA 3′UTR downregulating this reactivation factor, resulting in the reduction of KSHV reactivation [194]. Additionally, two other cellular miRNAs were able to target RTA 3′UTR (*i.e.*, hsa-miR-498 and hsa-miR-320d), promoting KSHV latency by repressing the RTA [195]. Interestingly, these two miRNAs are downregulated by HSV-1 infection, providing another mechanism modulating the KSHV lytic/latent cycles. Nevertheless, this kind of mechanism imposes the coexistence of both viruses in the same body compartment, which is not obvious, at least in the case of HSV-1 and KSHV, except during occasional HSV-1 viremia [195].

## 7. Conclusions

Interactions between virus and host are intricate, and miRNAs add yet another layer of complexity to them. In addition to encoding miRNAs, viruses can also deregulate cellular miRNAs to facilitate infection. By up- or downregulating key cellular miRNAs, viruses alter cellular gene expression to achieve similar results to those of viral miRNA targeting. Viral transcripts can also be targeted by cellular miRNAs with different outcomes. Generally, these cellular miRNAs repress viral gene expression, resulting in decreased viral replication.

Members of the *Herpesviridae* family are relevant examples of this complex and dynamic process where cellular and viral factors interact, resulting in lifelong viral infection. By targeting cellular mRNAs, herpesvirus miRNAs were reported to regulate genes related with immune response, apoptosis, cell cycle control, cell differentiation, and intracellular traffic. As many of these altered pathways are of the utmost importance for cellular homeostasis, their alterations largely contribute to viral pathogenesis.

The most noteworthy pathological event triggered by viral miRNAs is oncogenesis. In fact, oncogenic viruses such as EBV and KSHV/HHV-8 encode viral miRNAs that can be directly linked with the development of malignancies. Remarkably, although having different sequences, some EBV- and KSHV-encoded miRNAs (e.g., ebv-miR-BART2-5p and kshv-miR-K12-7) are able to repress identical targets (*i.e.*, MICB), thus contributing to a similar outcome (*i.e.*, reduced NK cell recognition and immune evasion). Moreover, while EBV induces hsa-miR-155 expression, KSHV encodes an orthologue for the same oncomir. Therefore, despite being evolutionarily distant, EBV and KSHV evolved separately and developed distinct strategies to achieve similar results: an establishment of lifelong infection, immune evasion, and ultimately host cell transformation.

This exciting new field may hold some answers as to why some viruses such as herpesvirus are so successful in infecting and establishing lifelong infections in human hosts. It could also help us decipher the molecular mechanisms underlying some important pathologies derived from herpesvirus infection, such as cancer.

This review has focused the interactions between herpesviruses, human host, and miRNAs; a word of caution should be added: the interplay between viral infection and miRNAs constitutes a field where part of the information relies on putative or indirect interactions. These interactions potentially dictate up- or downregulation of cell proteins that are key players in distinct cell pathways. However, miRNA-mRNA interactions are more complex than the mere base pairing mechanism between the seed region of the miRNA and the sequence of its target mRNA [51,196,197]. The prediction of such binding sites relies on algorithms based on base-pair complementarity but the potencial changes in the target gene expression induced by miRNAs imposes powerful and reliable experimental methods. *In vitro* studies based on the overexpression or inhibition of the miRNA of interest followed by the analysis of putative affected genes, although useful to confirm the computational miRNA target prediction, could yield false positive results, thus imposing the development of new strategies and more accurate experimental approaches.

Although not the subject of this review, it is important to emphasize that, during the last few years, several techniques (e.g., CLASH, RIP-CHIP, HITS-CLIP, PAR-CLIP, and proteomics analysis) have been used to functionally validate the targets of several viral miRNAs, particularly for those encoded by herpesviruses [94,183,198,199,200,201,202,203,204,205]. These new technologies allow unprecedented and largely unbiased views into miRNAs-mediated regulation of gene expression in virus-infected cells. In addition, it is of paramount importance that studies using *in vivo* models of infection are performed in order to confirm data obtained in *in vitro* experiments [206,207]. Notably, in the context of herpesvirus infection, there is little evidence from *in vivo* data regarding the role of viral-encoded miRNAs in the regulation of latent/lytic cycles of infection [62,208], either because they are context-dependent or due to insufficient sensitivity of the detection assay. Undoubtedly, further studies using different approaches and technologies are required towards the clear definition of miRNAs targetome and their functional relevance in viral infection, latency, and pathogenesis.

Finally, despite our limited knowledge, it is obvious that miRNAs constitute a potential therapeutic approach to viral infections and their pathological consequences. In fact, some advances have already been made in the development of therapeutic miRNAs against hepatitis C virus infection [108].

## Figures and Tables

**Table 1:** MicroRNAs (EBV- or cell-encoded) with potential role in EBV infection and pathogenesis.

miRNA		Target *	Predicted Role	References
**EBV-encoded** **(ebv-miR-)**	BHRF1-3	CXCL11 (c)	Immune evasion	[121]
	BART1	BIM (c)	Inhibits apoptosis	[122]
	BART1-3p	Caspase-3 (c)	Inhibits apoptosis	[123]
	BART1-5p	LMP1 (v)	Immune evasion	[120]
	BART2-5p	MICB (c)	Immune evasion	[124]
	BART3	BIM (c)	Inhibits apoptosis	[122]
	BART3-5p	DICE1 (c)	Cell transformation and proliferation	[125]
	BART5-5p	PUMA (c)	Inhibits apoptosis	[126]
	BART6-5p	Dicer (c)	Modulates biogenesis of microRNAs	[127,128]
	BART7	APC (c)	Cell transformation and proliferation	[129]
	BART9	BIM (c)	Inhibits apoptosis	[122]
	BART11	BIM (c)	Inhibits apoptosis	[122]
	BART12	BIM (c)	Inhibits apoptosis	[122]
	BART15	NLRP3 (c)	Immune evasion	[130]
	BART16	LMP1 (v)	Immune evasion	[130]
		TOMM22 (c)	Inhibits apoptosis	[122]
		Caspase-3 (c)	Inhibits apoptosis	[123]
	BART17-5p	LMP1 (v)	Immune evasion	[120]
	BART19-3p	WIF1 (c)	Cell transformation and proliferation	[129]
		APC (c)	Cell transformation and proliferation	[129]
	BART20-5p	BAD (c)	Inhibits apoptosis	[131]
**Cell-encoded** **(hsa-miR-)**	let-7a	Dicer (c)	Modulates biogenesis of microRNAs	[132]
	200b	ZEB1, ZEB2 (c)	Modulates latent/lytic infection	[133,134]
	429	ZEB1, ZEB2 (c)	Modulates latent/lytic infection	[133,134]

* Detailed information on potential targets is mentioned in the text. Abbreviations used: APC—adenomatous polyposis coli; BAD—BCL2-associated agonist of cell death; BIM—BCL2 interacting mediator of cell death; CXCL11—C-X-C motif chemokine 11; DICE1—Deleted in cancer 1; LMP—Latent membrane protein; MICB—major histocompatibility complex class-I related chain B; NLRP3—NLR family, pyrin domain containing 3; PUMA—p53-upregulated modulator of apoptosis; TOMM22—translocase of outer mitochondrial membrane 22 homolog (yeast); ZEB1/ZEB2—Zinc finger E-box-binding homeobox (1 or 2); (c) Cellular gene; (v) Viral gene.

**Table 2:** MicroRNAs with potential role in KSHV infection and pathogenesis.

KSHV-Encoded miRNA (kshv-miR-)	Target *	Predicted Role	References
**K12-1**	MICB	Immune evasion	[124]
	p21	Oncogenesis	[164]
	IκBα	Cell survival	[165]
		Modulates latent/lytic infection	[166]
	Caspase 3	Cell survival	[167]
	xCT expression	Facilitates viral entry	[168,169,170,171]
**K12-3**	C/EBPβ	Immune evasion	[168,158]
		Cell survival	[168,158]
		Oncogenesis	[168,158]
	Caspase 3	Cell survival	[167]
	NFIB	Modulates latent/lytic infection	[172]
**K12-4**	RBL2	Modulates latent/lytic infection	[173]
	Caspase 3	Cell survival	[167]
**K12-5**	BCLAF1	Modulates latent/lytic infection	[174]
	MYD88	Immune evasion	[175]
**K12-6**	xCT expression	Facilitates viral entry	[168,169,170,171]
**K12-7**	C/EBPβ	Immune evasion	[168]
		Cell survival	[168]
		Oncogenesis	[168]
	KSHV ORF50	Modulates latent/lytic infection	[176,177]
**K12-9**	IRAK1	Immune evasion	[175]
	xCT expression	Facilitates viral entry	[168,169,170,171]
	KSHV ORF50	Modulates latent/lytic infection	[176,177]
	BCLAF1	Modulates latent/lytic infection	[174]
**K12-10**	TWEAKR	Immune evasion	[178]
		Cell survival	[178]
	TGFBR2	Cell survival	[166]
		Oncogenesis	[166]
**K12-11**	IKKε	Immune evasion	[179]
		Modulates latent/lytic infection	[179]
	C/EBPβ	Immune evasion	[180]
		Cell survival	[180]
		Oncogenesis	[180]
	SMAD5	Cell survival	[181]
		Oncogenesis	[181]
	xCT expression	Facilitates viral entry	[168,169,170,171]

* Detailed information on potential targets is mentioned in the text. Abbreviations used: BCLAF1—BCL2-associated transcription factor 1; C/EBPβ—CCAAT/enhancer binding protein beta; IκBα—nuclear factor of kappa light polypeptide gene enhancer in B-cells inhibitor, alpha; IKKε—IκB kinase epsilon; IRAK1—interleukin 1 receptor-associated kinase 1; KSHV ORF50—Kaposi’s sarcoma-associated herpesvirus open reading frame 50; MICB—major histocompatibility complex class-I related chain B; MYD88—myeloid differentiation primary response 88; NFIB—nuclear factor I/B; p21—cyclin-dependent kinase inhibitor 1A; RBL2—retinoblastoma-like protein 2; SMAD5—SMAD family member 5; TGFBR2—transforming growth factor, beta-receptor II; TWEAKR—tumor necrosis factor-like weak inducer of apoptosis receptor; xCT—solute carrier family 7 (anionic amino acid transporter light chain, xC- system), member 11.

## Abbreviations

The following abbreviations are used in this manuscript:

AIDS: Acquired immunodeficiency syndrome
APC: Adenomatous polyposis coli
ATP5B: Mitochondrial ATP synthase subunit beta
ATP6V0CP1: ATPase, H^+^ transporting, lysosomal 16 kDa, V0 subunit C pseudogene 1
BACH-1: BTB and CNC homology 1, basic leucine zipper transcription factor 1
BAD: BCL2-associated agonist of cell death
BAX: BCL2-associated X Protein
BCL2: B-cell CLL/lymphoma 2
BCL6: B-cell CLL/lymphoma 6
BCLAF1: BCL2-associated transcription factor 1
BIM: BCL2 interacting mediator of cell death
BL: Burkitt’s lymphoma
BRCC3: BRCA1/BRCA2-containing complex, subunit 3
C/EBPβ: CCAAT/enhancer binding protein β
CCL5: (C-C Motif) Ligand 5
CCNE2: Cyclin E2
CD147: Collagenase stimulatory factor
CDC42: Cell division control protein 42
CLASH: Cross-linking ligation and sequencing of hybrids
CXCL11: C-X-C motif chemokine 11
CXCR4: Chemokine (C-X-C motif) receptor 4
DGCR8: DiGeorge syndrome critical region gene-8
DICE1: Deleted in cancer 1
DLBCL: Diffuse large B-cell lymphoma
DLL4: Delta-Like 4 (Drosophila)
DNA: Deoxyribonucleic acid
EBNA: Epstein–Barr nuclear antigen
EBV: Epstein–Barr Virus
EID1: EP300 interacting inhibitor of differentiation 1
eIF4A1: Eukaryotic initiation factor 4A1
ERAP1: Endoplasmic reticulum aminopeptidase 1
ERC: Endocytic recycling compartment
FLICE: Fas-associated death domain-like interleukin-1 β-converting enzyme
GC: Gastric carcinoma
H3F3B: H3 histone, family 3B
HCMV: Human cytomegalovirus
HCV: Hepatitis C virus
HD: Hodgkin’s disease
HITS-CLIP: High-throughput sequencing of cross-linked immuno-precipitation
HIV-1: Human Immunodeficiency Virus 1
HSV-1: Herpes Simplex Virus type 1
HSV-2: Herpes Simplex Virus type 2
ICP0: HSV-infected cell polypeptide 0
ICP34.5: HSV-infected cell polypeptide 34.5
ICP4: HSV-infected cell polypeptide 4
IκBα: Nuclear factor of kappa light polypeptide gene enhancer in B-cells inhibitor, alpha
IKKε: IκB kinase epsilon
IL-18: Interleukin 18
IL-1α: Interleukin 1α
IL-1β: Interleukin 1β
IL-6: Interleukin-6
IRAK1: Interleukin 1 receptor-associated kinase 1
IRF1: Interferon regulatory factor 1
KLHL24: Kelch-Like Family Member 24
KS: Kaposi’s sarcoma
KSHV: Kaposi’s sarcoma-associated herpesvirus
LANA: Latency-associated nuclear antigen
LAT: HSV latency-associated transcript
LMP: Latent membrane protein
MAF: v-Maf avian musculoaponeurotic fibrosarcoma oncogene homolog
MAPRE2: Microtubule-associated protein, RP/EB family, member 2
MCD: Multicentric Castleman’s disease
MCP-1: Monocyte chemoattractant protein 1
MHC-I: Major histocompatibility complex, class I
MICA: Major histocompatibility complex class-I related chain A
MICB: Major histocompatibility complex class-I related chain B
miRISC/RISC: miRNA-induced silencing complex
miRNA: microRNA
mRNA: Messenger RNA
MYD88: Myeloid differentiation primary response 88
NDRG1: N-Myc Downstream-Regulated Gene 1
NFIB: Nuclear factor I/B
NFκB: Nuclear factor kappa-light-chain-enhancer of activated B cells
NKG2D: Killer cell lectin-like receptor subfamily K, member 1
NKTL: NK/T-cell lymphoma
NLRP3: NLR family, pyrin domain containing 3
NPC: Nasopharyngeal carcinoma
ORF: Open reading frame
p21: Cyclin-dependent kinase inhibitor 1A
PAR-CLIP: Photoactivatable-ribonucleoside-enhanced crosslinking and immunoprecipitation
PEL: Primary effusion lymphoma
pri-miRNA: Primary-microRNA
PTEN: Phosphatase and tensin homolog
PTLD: Post-transplant lymphoma
PUMA: p53-Upregulated modulator of apoptosis
RAB11A: RAS-related GTP-binding protein 11A
RAB5C: RAS-related GTP-binding protein 5C
Rbl2: Retinoblastoma-like protein 2
RIP-CHIP: RNA-binding protein immunoprecipitation-microarray (Chip)
RNA: Ribonucleic acid
RNAi: RNA interference
RTA: R transactivator
siRNA: Small interference RNA
SMAD5: SMAD family member 5
SNAP23: Synaptosomal-associated protein, 23 kDa
TGF-β: Transforming growth factor beta
TGFBR2: Transforming growth factor, β-receptor II
TIMP3: Tissue inhibitors of metalloprotease 3
TLR2: Toll-like receptor 2
TNF-α: Tumor necrosis factor α
TOMM22: Translocase of outer mitochondrial membrane 22 homolog (yeast)
TR: Terminal repeat
TWEAK: TNF-related weak inducer of apoptosis
TWEAKR: TWEAK receptor
UTR: Untranslated region
VAC: Virion assembly compartment
VAMP3: Vesicle-associated membrane protein 3
WIF1: Wnt inhibitor factor 1
xCT: Solute carrier family 7 (anionic amino acid transporter light chain, xC-system), member 11
ZEB1/ZEB2: Zinc finger E-box-binding homeobox (1 or 2)
